# A Febrile Infant With Abdominal Erythema and Irritability

**DOI:** 10.1177/00099228231162413

**Published:** 2023-03-25

**Authors:** Jaron A. Smith, Jennifer Tiller, Elizabeth Lagomarsino, Joseph Murphy

**Affiliations:** 1 Department of Pediatrics, Division of Pediatric Emergency Medicine, UT Southwestern, Dallas, TX, USA; 2 Department of Radiology, Division of Pediatric Radiology, UT Southwestern, Dallas, TX, USA; 3 Department of Surgery, Division of Pediatric Surgery, UT Southwestern, Dallas, TX, USA

## Case Report

A 37-day-old male was evaluated in the emergency department for 24 hours of fever to 102°F, abdominal erythema, and irritability. The patient was also reported to have 10 days of watery, gray stools on an appropriate diet of generic formula and breastmilk. The patient was the product of a full-term pregnancy and a C-section delivery due to breech positioning with no perinatal complications.

**Figure 1.**
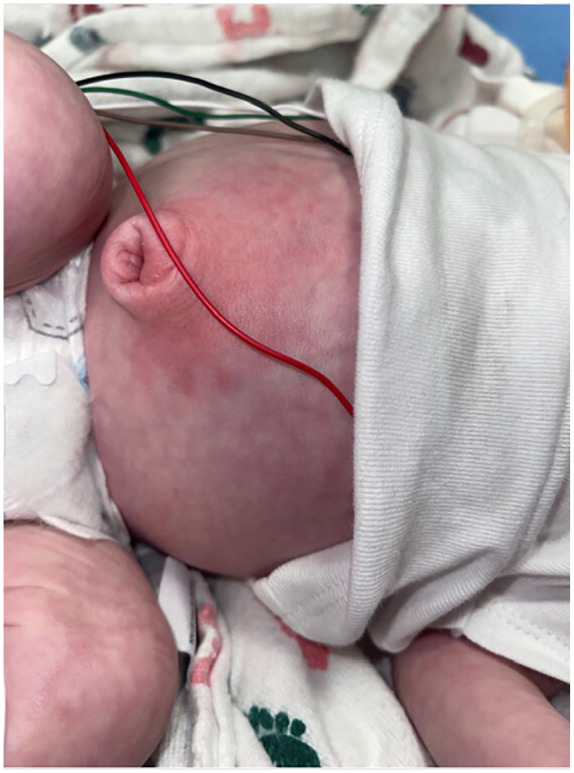
Physical exam of patient’s abdomen showing periumbilical erythema and mild umbilical hernia. The patient’s abdomen was also distended and tender to palpation.

On initial assessment, the patient was febrile to 38.6°C, tachycardic to 190 bpm, tachypneic to 70 breaths/minute with normal oxygen saturations and blood pressure. He was fussy and difficult to console, and he had obvious erythema of the umbilical and periumbilical region (Figure 1). The abdomen was diffusely tender to palpation with gross distension. A small umbilical hernia was irreducible with patient straining.

Pertinent laboratory findings included a normal complete blood count and complete metabolic profile, C-reactive protein of 10.7 mg/dL (ref.: 0.0-1.0 mg/dL), and procalcitonin of 1.47 ng/mL (ref.: <0.05 ng/mL). Urinalysis was unremarkable, urine and blood cultures were drawn, and an attempted lumbar puncture was unsuccessful.

**Figure 2.**
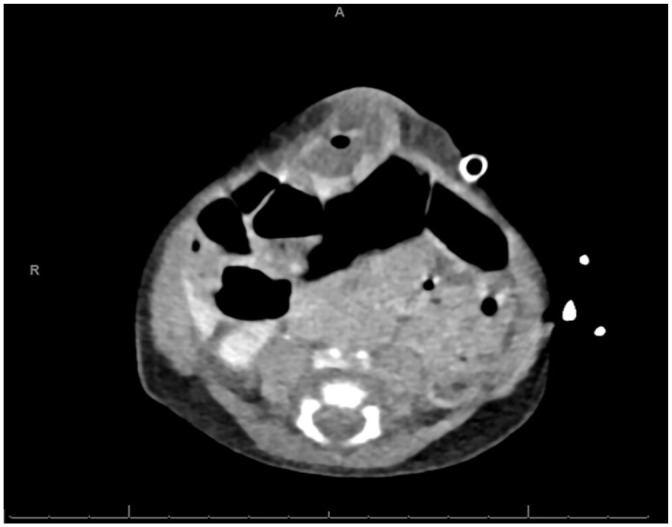
Axial view of abdominal computed tomography showing hypodense regions in the anterior abdomen and a small air pocket within those regions.

Plain films demonstrated diffuse gaseous distension of bowel throughout the abdomen without dilated loops of bowel. Intraluminal gas extended to the rectum. No appreciable free intraperitoneal air, pneumatosis, or portal venous gas was noted. Abdominal ultrasound showed hyperemic soft tissue with surrounding inflammatory stranding and a small amount of fluid with mild hyperemia of the bowel near the site of the herniation suggestive of an incarcerated umbilical hernia without strangulation. An abdominal computed tomography (CT) (Figure 2) suggested a central abdominal wall hernia containing loops of small bowel with associated mild mucosal hyper-enhancement and thickening, potentially concerning for strangulation/incarceration, but without evidence of bowel obstruction. Alternatively suggested was a small fluid collection related to an infected umbilicus.

Piperacillin/tazobactam (80 mg/kg), vancomycin (20 mg/kg), and normal saline (20 mL/kg) were given in preparation of operative intervention for a presumed incarcerated umbilical hernia and resulting intra-abdominal infection.

## Discussion

### Hospital Course

During operative exploration, the periumbilical subcutaneous tissue and surrounding skin was inflamed and friable, and the umbilical stalk was woody and friable, suggesting omphalitis. No umbilical hernia with strangulated viscera was identified; however, an intraperitoneal abscess was found abutting the underside of the anterior abdominal wall at the base of the umbilical stalk. Extensive debridement of all associated fibrinopurulent tissues revealed a perforated Meckel’s diverticulum approximately 10 cm proximal to the ileocecal valve abutting the abscess cavity. A small defect at the base of the umbilical stalk where the abscess had eroded through the anterior abdominal wall was repaired. Pathology report would confirm a Meckel’s diverticulum with gastric heterotopia, transmural inflammation, focal necrosis, and acute serositis.

Postoperative care for sepsis required admission to the pediatric intensive care unit where the patient was treated with ceftriaxone (50 mg/kg) daily and metronidazole (10 mg/kg) every 8 hours concurrently for 5 days, then metronidazole alone for an additional 6 days. Blood and urine cultures had no growth, and abscess cultures were unable to be obtained. Total parenteral nutrition was required for 7 days, after which enteral feeds were initiated and well tolerated. The patient was discharged in good condition. One year from surgery, the patient is growing and developing appropriately.

### Discussion of Case and Literature

A ruptured Meckel’s diverticulum in infancy is rare,^1-4^ as is omphalitis occurring outside of the newborn period.^5,6^ However, to our knowledge, this is the first reported case of a ruptured Meckel’s diverticulum leading to omphalitis in infancy outside of the newborn period.

Omphalitis is an infection of the umbilical stump, the umbilicus, and/or the surrounding tissues, and is distinct from peritonitis, which is infection of the peritoneum, or abdominal wall.^7^ Omphalitis typically presents as a superficial cellulitis. Infection is typically an external source, associated with cutting of the umbilical cord. Infections are often polymicrobial, and common pathogens include staphylococcal and streptococcal species, *E. coli*, *Klebsiella*, and anaerobes.^8^ These track internally from the severed external umbilical stump, then infect the nearby soft tissues.^5,7,9^ Because there was no guidance provided by culture results in our patient, empiric therapy was aimed at coverage of these organisms.

Omphalitis can rapidly progress,^5,9^ has high morbidity,^5,6,9^ and mortality ranges 7% to 15%,^5^ thus, early recognition is critical. Because of the cause associated with cutting and healing of the umbilical cord, it is uncommon for omphalitis to occur outside the newborn period.^5-7,10^ In these cases, the differential should be broadened, and internal sources that then infect the umbilical stump and surrounding tissues need to be considered, including infected remnants of prenatal structures associated with the umbilicus.^10,11^ As such, advanced imaging is typically required to identify the source, and surgical management is often warranted.^5,7^

A perforated or ruptured Meckel’s diverticulum is one of these rare causes of nonneonatal omphalitis. A Meckel’s diverticulum is a true diverticulum that involves all 3 layers of the bowel wall.^1,2,12^ It is a vestigial remnant of the omphalomesenteric duct, also known as the vitelline duct or yolk stalk.^1,12^ Diagnosis of a Meckel’s diverticulum is typically made only when pursued due to clinical complications, and is challenging since CT and ultrasound may conflate a diverticulum as normal bowel. A technetium-99m pertechnetate scan is considered the gold standard, but is not widely available, and is specific only to the Meckel’s diagnosis.^2^ Ruptured Meckel’s diverticulum is itself an uncommon complication.^1,4^ Although there are case reports and series occurring in the neonatal period^4^ and in children older than 12 months,^2,13^ the incidence occurring in nonnewborn infancy has only been reported twice since 1975.^1,14^ Presenting symptoms are variable, but common complications include bloody stool, bowel obstruction, sepsis, peritonitis, and much less commonly, as reported in our case, omphalitis.^1,2,15^

### Final Diagnosis

Ruptured Meckel’s diverticulum with resulting umbilical stalk abscess and omphalitis.

## Conclusion

In summary, omphalitis outside of the neonatal period is rare and requires consideration of internal causes, including infections and complications of primitive remnants. Complications of Meckel’s diverticulum are numerous, but less commonly known is perforation, which can then lead to local and systemic infection, including omphalitis and sepsis. Surgical consultation was required, and despite advanced imaging, the final diagnosis was not made until operative management, highlighting the need to involve surgery early if either of these diagnoses is considered.

### Educational Objectives

- Omphalitis outside of the neonatal period is a rare entity, and likely to have an internal cause that must be evaluated with the assistance of imaging and surgical consultation.
- A ruptured Meckel’s diverticulum is a rare complication of a diagnosis that already requires a high index of suspicion.

## Author Contributions

Dr. Smith authored the original draft and all subsequent edits. Drs. Tiller, Lagomarsino, and Murphy provided mentorship, specialty-specific insight, and reviewed and edited all drafts.
